# A microRNA signature of response to erlotinib is descriptive of TGFβ behaviour in NSCLC

**DOI:** 10.1038/s41598-017-04097-7

**Published:** 2017-06-23

**Authors:** Madeline Krentz Gober, James P. Collard, Katherine Thompson, Esther P. Black

**Affiliations:** 1 Department of Pharmaceutical Sciences, College of Pharmacy, University of Kentucky, Lexington, KY 40536-0596 USA; 2 Department of Statistics, College of Arts and Sciences, University of Kentucky, Lexington, KY 40536-0082 USA

## Abstract

Our previous work identified a 13-gene miRNA signature predictive of response to the epidermal growth factor receptor (EGFR) inhibitor, erlotinib, in Non-Small Cell Lung Cancer cell lines. Bioinformatic analysis of the signature showed a functional convergence on TGFβ canonical signalling. We hypothesized that TGFβ signalling controls expression of the miRNA genes comprising an erlotinib response signature in NSCLC. Western analysis revealed that TGFβ signalling via Smad2/3/4 occurred differently between erlotinib-resistant A549 and erlotinib- sensitive PC9 cells. We showed that TGFβ induced an interaction between Smad4 and putative Smad Binding Elements in PC9. However, qRT-PCR analysis showed that endogenous miR-140/141/200c expression changes resulted from time in treatments, not the treatments themselves. Moreover, flow cytometry indicated that cells exited the cell cycle in the same manner. Taken together these data indicated that the miRNA comprising the signature are likely regulated by the cell cycle rather than by TGFβ. Importantly, this work revealed that TGFβ did not induce EMT in PC9 cells, but rather TGFβ-inhibition induced an EMT-intermediate. These data also show that growth/proliferation signals by constitutively-activated EGFR may rely on TGFβ and a possible relationship between TGFβ and EGFR signalling may prevent EMT progression in this context rather than promote it.

## Introduction

Lung cancers are frequently diagnosed in later stages of disease progression with few treatment options available for patients. In the last decade, a number of targeted therapies have been developed against impactful oncogenic targets in lung cancer (e.g. EGFR, ALK, and ROS), but many tumours either lack an actionable oncogenic mutation or harbour an inherent resistance mutation (e.g. KRAS). Therefore, most patients receive a cytotoxic agent to which they may not respond^1, 2^. Unfortunately, many patients with a targetable mutation eventually develop resistance to targeted therapy enforcing the need to couple or stage therapies to combat resistance.

Genome scale sequencing and gene expression technologies have provided scientists and clinicians the tools to gather increasingly more specific insight on tumour heterogeneity thereby allowing for tumour-specific therapeutic decisions to be made. While the ability to characterize tumours at this level has revolutionized the concept of personalized cancer care, the breadth of information presents the dilemma of how to interpret which molecular characteristics are biologically relevant for treatment decisions. Recently, The Cancer Genome Atlas (TCGA) conducted genomic, transcriptomic, and proteomic profiling of 230 lung adenocarcinomas revealing that 73% of the tumours studied showed activation of the Ras/Raf cascade downstream of a Receptor Tyrosine Kinase (RTK) at the level of genomic alterations and gene expression, but only a subset of those tumours showed aberrant activation of this cascade at the protein level^3^. This observation underscores the diversity within and between tumours reinforcing the need for multivariate predictors of drug response to overcome the failings of single biomarker methods of response prediction.

One of the more commonly targeted oncogenic RTKs in Non-Small Cell Lung Cancers (NSCLC) is the Epidermal Growth Factor Receptor (EGFR). The EGFR inhibitor, erlotinib, is indicated for use in patients harbouring an EGFR-activating mutation (10–15% of patients) and is contraindicated for use in patients with mutated KRAS (25–30% of patients)^4^. Using only these two markers to assign erlotinib treatment in NSCLC has yielded results that are modest at best^5^. To augment the short-comings of KRAS and EGFR mutation status as the sole predictive metric, this lab showed that microRNA (miRNA) expression patterns in different cell lines could predict erlotinib resistance, reporting that a 13-miRNA signature could be used for these purposes^6^. Our 13-miRNA gene signature of response is not only able to stratify NSCLC cells and tumour samples into erlotinib- sensitive and –resistant groups, but was also able to discriminate between primary and metastatic lesions. Understanding why the expression of these small RNA molecules can distinguish response to anti-EGFR therapy and discriminate metastatic lesions has implications for both prognostic and predictive clinical applications.

MicroRNA are non-coding, small, RNA that regulate gene expression by pairing with complementary mRNA resulting in translation inhibition or degradation of the mRNA^7^. miRNA play a role in a number of biological processes (e.g. growth, differentiation, and proliferation), so it is not surprising that endogenous expression levels are deregulated in cancer^8^. Bioinformatic analysis of the 13-gene miRNA signature showed that many of the proposed target genes functionally converge on the TGFβ signalling pathway^6^. For this study, we specifically focused on signature members miR-140, -141, and -200c due to their opposing expression between erlotinib- sensitive and –resistant cell lines. The miR-200 family, including miR-200c and −141, is well-characterized for preventing EMT onset by targeting transcription factors (e.g. Zeb1 and 2) responsible for suppressing expression of epithelial characteristics, such as the E-cadherin (E-cad) adhesion proteins^9–12^. High expression of these two miRNA correlate with erlotinib-sensitivity in the 13-miRNA signature. Conversely, miR-140 is highly expressed in erlotinib-resistant cells and is predicted to target the TGFβ receptor and Smad2^6, 13^. Importantly, these data demonstrate that opposing expression profiles and activities are necessary for EMT.

The TGFβ signalling pathway is well documented for its role in the induction and potentiation of the mesenchymal phenotype in tumour cells^14^. TGFβ is a ubiquitous cytokine that is active in a number of cell processes, and many of cell types secrete the ligand and express the receptors to bind it^15^. Activation of TGFβ signaling is accomplished by TGFβ ligands binding to the extracellular domain of TGFβII receptors. This allows it to recruit the TGFβI receptor and then bind a second pair of activated TGFβII/I receptors resulting in transautophosphorylation within the tetramer^16^.

TGFβ canonical signaling is mediated by Smads 2, 3, and 4, which bind to Smad Binding Elements (SBE) on DNA eliciting a transcriptional response^17^. TGFβ potentiates the Epithelial to Mesenchymal Transition (EMT) in some cancer cells by signalling through a variety of other non-canonical pathways including PI3K/AKT and MAPK/ERK^18^. Interestingly, several groups have noted that erlotinib sensitivity tends to correlate with the epithelial phenotype^19^. Since TGFβ upregulates genes responsible for the activation of the EMT program^20^, and because the miRNA signature is capable of stratifying between primary and metastatic lesions *ex vivo* ^6^, we hypothesize that TGFβ supports differential expression of the signature miRNA between erlotinib-resistant and –sensitive NSCLC.

## Results

### Most signature miRNA promoters contain Smad Binding Elements

**Figure 1:**
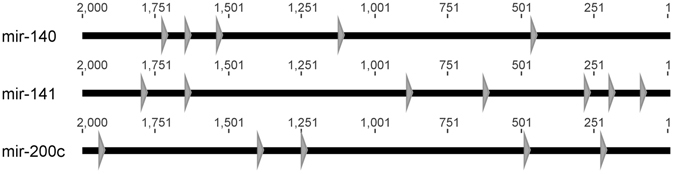
Signature microRNA genes contain SBE elements. Promoter analysis was conducted using the ChipMAPPER algorithm^21, 22^. microRNA genes -140, -141, and -200 contain putative SBE elements as represented by the triangle with conservative E-values less than or equal to 25 and a score greater than 3.0.

The promoter of each of the 13 signature miRNA was analysed using chipMAPPER^21, 22^ for putative SBEs^17, 23^. Predicted SBEs were retained if they had conservative E-values (≤25) and a score greater than 3.0. SBEs matching these criteria were found in the promoter regions of twelve of the thirteen signature miRNA (Supplemental Figure 1). The three signature miRNA genes we focused on in this study (mir-140, -141, and -200c) have multiple predicted SBEs within -2000 base pairs of transcriptional start site (Fig. 1)^17, 24, 25^.

The activity of complexes containing Smad2 and -3 along with the DNA-binding member, Smad4, have been shown to have both positive and negative effects on transcription^26, 27^. Since the signature miRNA are differentially expressed among cell lines, the majority of their promoters contain putative SBEs, and the known dual behaviour of TGFβ activity on gene expression, we hypothesized that the canonical TGFβ signalling pathway likely controls in the opposing expression levels of signature miRNA between erlotinib-resistant and - sensitive NSCLC lines.

### TGFβ-mediated Smad signalling has an opposing phenotype in erlotinib-resistant and -sensitive cell lines

A549 and PC9 cell lines were selected as representative NSCLC cell lines due to their opposing erlotinib responses and opposing expression levels of the 3 candidate miRNA genes. A549 are inherently erlotinib- resistant because they harbor a KRAS activation mutation, and PC9 are erlotinib-sensitive treatment because they contain an activating exon 19 deletion in EGFR^28^.

**Figure 2:**
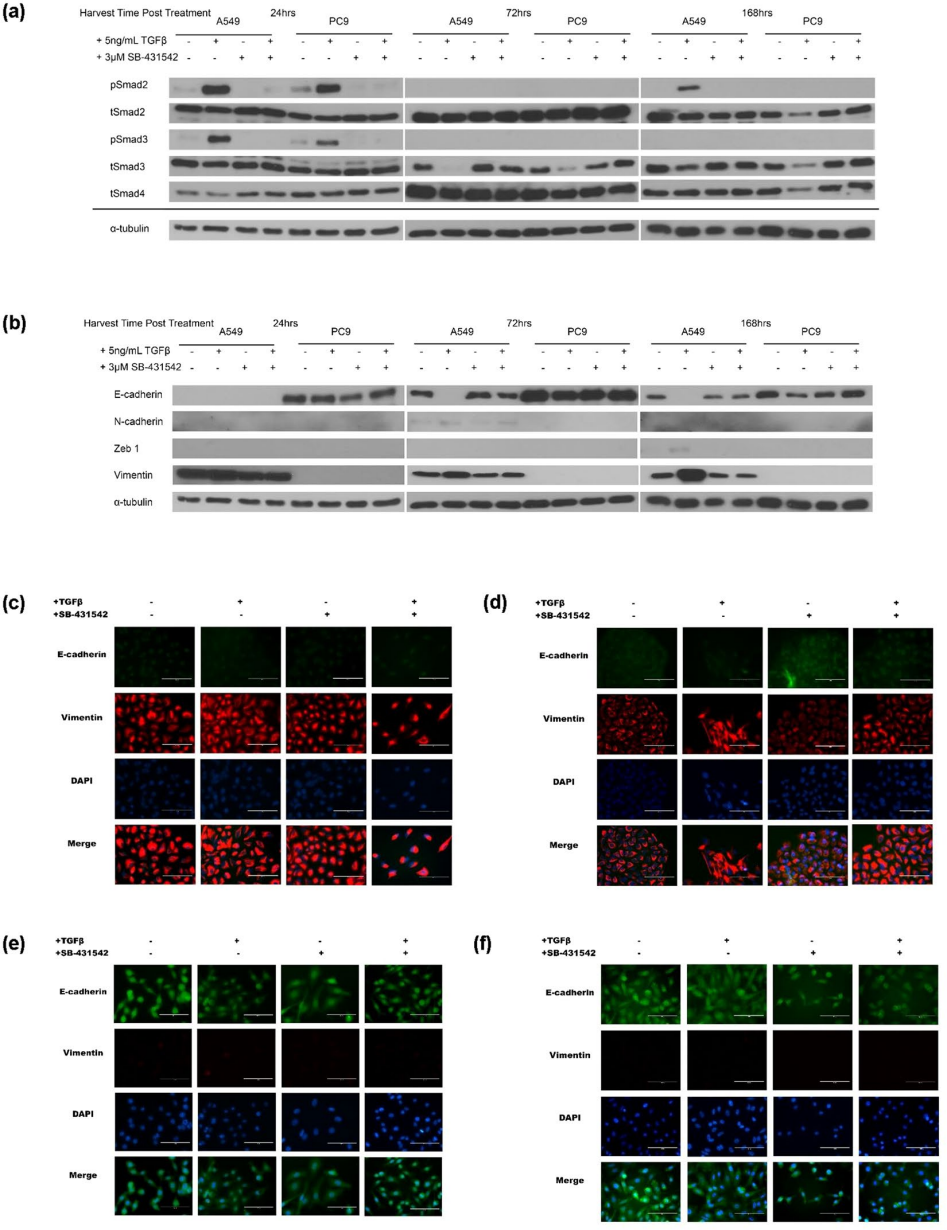
Total Smad expression, Smad activation and EMT program marker expression varies with TGFβ or inhibitor treatment. A549 and PC9 cells were plated, treated and harvested as described. Proteins were visualized by western blotting. α-tubulin levels are representative controls. (**a**) Profiling of Smad family member expression and activation across time demonstrates changes in TGFβ canonical signalling. (**b**) EMT protein markers demonstrate program initiation and progression among treatment conditions. (**c**) A549 cells treated for 24 hours for E-cadherin and Vimentin expression by immunofluorescence (**d**) A549 cells treated for 7 days for E-cadherin and Vimentin expression by immunofluorescence (**e**) PC9 cells treated for 24 hours for E-cadherin and Vimentin expression by immunofluorescence (**f**) PC9 cells treated for 7 days for E-cadherin and Vimentin expression by immunofluorescence.

We first examined the expression and activation of the Smad molecules, Smad2, Smad3, and Smad4, after treatment with exogenous TGFβ ligand, an inhibitor of TGFβRII, SB-431542, or the combination in these cell lines (Fig. 2a) by western blot to determine if these effectors could be responsible for signature miRNA regulation. In both A549 and PC9 after 24 hours of treatment, pSmad2 and pSmad3 levels seem elevated in cells treated with TGFβ, and the effect was diminished in cells treated with SB-431542 or the combination of SB- 431542 and TGFβ. Total Smad2, Smad3 and Smad4 levels appear to be consistently expressed across treatments at 24 h. There were no obvious levels of pSmad2 or pSmad3 in either cell line or in any treatment condition at the 72-hour treatment time point. Total Smad2 and Smad4 levels appear to be consistently expressed in both cell lines across both treatments. However, in both cell lines, tSmad3 levels were diminished in cultures treated with TGFβ.

At 168 hours, pSmad2 levels were seen only in A549 treated with TGFβ. Phospho-Smad3 levels were not observed in either line at 168 hours. tSmad2, tSmad3, and Smad4 appear diminished in PC9 treated with TGFβ alone, and this phenotype was not observed in any other condition. A549 demonstrated similar expression of total Smad molecules across all treatment conditions.

We observed the cyclical activation of Smad2 in A549 while activation of Smad3 was observed early following initial stimulation, but did not return. In PC9, a different phenotype emerged with diminished levels of all Smad2, -3, and -4 molecules by 168 hours. Taken together, these data suggest that the TGFβ canonical signals are managed differently in A549 and PC9.

### TGFβ treatment induces an EMT protein expression switch in A549 but not in PC9

Like many, we observed that A549 treated with TGFβ undergo a morphological change with treatment and appropriately activate R-Smad proteins - a phenotype consistent with EMT. PC9 cells did not undergo these changes with TGFβ treatment, but interestingly, PC9 cells treated with the TGFβ inhibitor displayed an EMT intermediate phenotype known as “Metastable” (Supplemental Figure 2)^29^. For this reason, we assessed a panel of EMT protein markers to determine if the morphological changes observed were indicative of EMT progression and correlated with signature miRNA endogenous expression changes.

A549 and PC9 were plated, treated, and harvested as described for protein measured by BCA assay prior to western blotting. Lysates were assessed for mesenchymal markers N-Cadherin (N-cad), Zeb1, and Vimentin as well as the epithelial marker, E-cadherin (E-cad) to confirm if the morphological changes were consistent with EMT occurring (Fig. 2b). As a comparison, we also profiled A549 and PC9 cells for E-cad and Vimentin expression by immunofluorescence at 24- and 168-hour (7 days) time points (Fig. 2c–f). mRNA levels of E-cadherin were examined in both cell lines at 24-, 72- and 168-hour time points to fully capture the change in expression of this epithelial marker across time points (Supplementary Figure 3).

In A549 cells, TGFβ treatment suppressed E-cad expression across each of the time points in the experiment, as expected. Conversely, Vimentin expression increased over the time course of TGFβ treatment. N-cad and Zeb1 appeared in the 72 and 168 hour time points respectively in TGFβ-treated A549 cells. The immunofluorescence profile of E-cad expression at the 24 hour and 7 day –treated timepoints in A549 cells was consistent with the levels observed by western analysis. Vimentin levels increased in A549 cells also mirrored the western blot results (Fig. 2c,d).

In PC9 cells, neither TGFβ treatment nor its inhibition altered E-cad expression or expression of the mesenchymal markers assessed. E-cad expression was consistent between the western and immunofluorescent assays. Vimentin expression was not observed by western or immunofluorescence assays (Fig. 2e,f). Since PC9 cells responded unexpectedly to treatment, as expected due of their epithelial nature, we sought to determine whether TGFβ directly regulated the expression of two candidate miRNA genes in both A549 and PC9 by assessing if Smad4 directly binds a shared putative SBE.

### TGFβ induces Smad4 binding to putative SBEs in the promoter of mir-141/200c in erlotinib-sensitive cells

To test the impact of the observed deregulation of R-Smad activity in A549 and PC9 cells on candidate miRNA expression, we asked whether Smad4 was directly binding the promoters of our miRNA genes. Smad4 is the only member of the canonical-Smad family with a nuclear localization signal, and others have shown that it is required for any active Smad complex to translocate into the nucleus to regulate transcription. Direct regulation of gene expression by TGFβ-activated Smad complexes is expected to occur within 24 hours of treatment^24^. For these reasons, we only tested Smad4 binding to the SBE locus after 24 hours of treatment by Chromatin Immunoprecipitation (ChIP).

**Figure 3:**
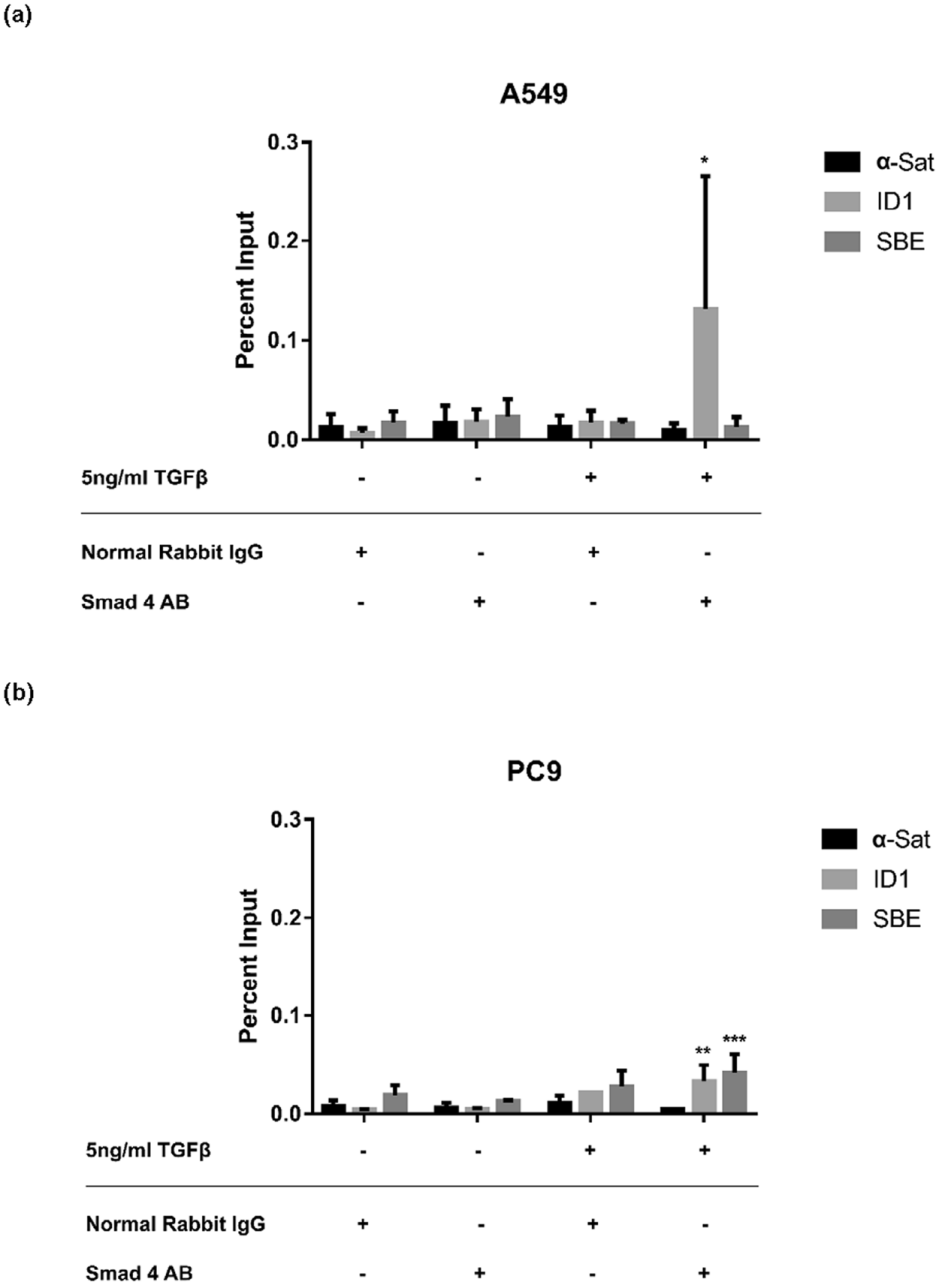
TGFβ induces Smad4 binding to SBEs in the promoter of mir-200/141 in PC9 cells. Chromatin immunoprecipitation was performed to identify whether a physical interaction between Smad4 and a predicted SBE locus in the shared promoter of mir-200c/-141 resulted from TGFβ treatment. Normal rabbit IgG served as the antibody negative control and α-Satellite primers as the negative PCR control. ID1 locus immunoprecipitation was the positive control for Smad4 binding. (**a**) In A549, positive Smad4-ID1 association is observed with TGFβ treatment, but an Smad4-SBE interaction is not. (**b**) In PC9, both Smad4-ID1 and Smad4-SBE interaction is observed. Significance was calculated using an unpaired t-test comparing TGFβ-treated cells and -untreated samples with the same primer set.

In A549 cells, TGFβ treatment induced a significant enrichment of the positive control, the ID1 promoter SBE, bound to Smad4 (p = 0.0171). The mir-141/-200c promoter region was not significantly enriched in A549 cells in any treatment or antibody combination (Fig. 3a). In PC9 cells, TGFβ treatment enriched both the positive control, ID1 (p = 0.0035), and the mir-141/-200c promoter containing the SBE locus (p = 0.0006), suggesting that Smad4 is bound to the shared promoter region in PC9 cells and not in A549 cells treated with TGFβ (Fig. 3b). This observation led us to ask whether the observed DNA interaction between the Smad4- containing complex and the SBE resulted in changes in endogenous levels of miR-141 or -200c.

### Time, not treatment, alters the expression of the candidate microRNAs

Activated Smad2 and -3 were present in both lines at 24 h post-TGFβ treatment and pSmad2 returned at 168 h after treatment in A549 cells. We have also shown that Smad4 is expressed in all conditions and binds mir-141/200c promoter at 24 h post-TGFβ treatment in PC9. For these reasons, we anticipated that differential expression of the signature miRNA genes would occur under these conditions as a result of TGFβ treatment. To explore this, A549 and PC9 cells were cultured and harvested as described and assessed for endogenous expression changes of three signature miRNA genes, miR-140 -141, and -200c by qRT-PCR. Importantly, these experiments were performed in 1%-serum media to minimize the impact of exogenous cytokines. We tested each of the three miRNA genes profiled in the conditions indicated here in both 1% serum and 10% serum treatment conditions to confirm that the changes observed are not due to serum levels. Importantly, miRNA expression does not significantly differ between the two serum levels for any of these three miRNA genes (Data not shown).

**Figure 4:**
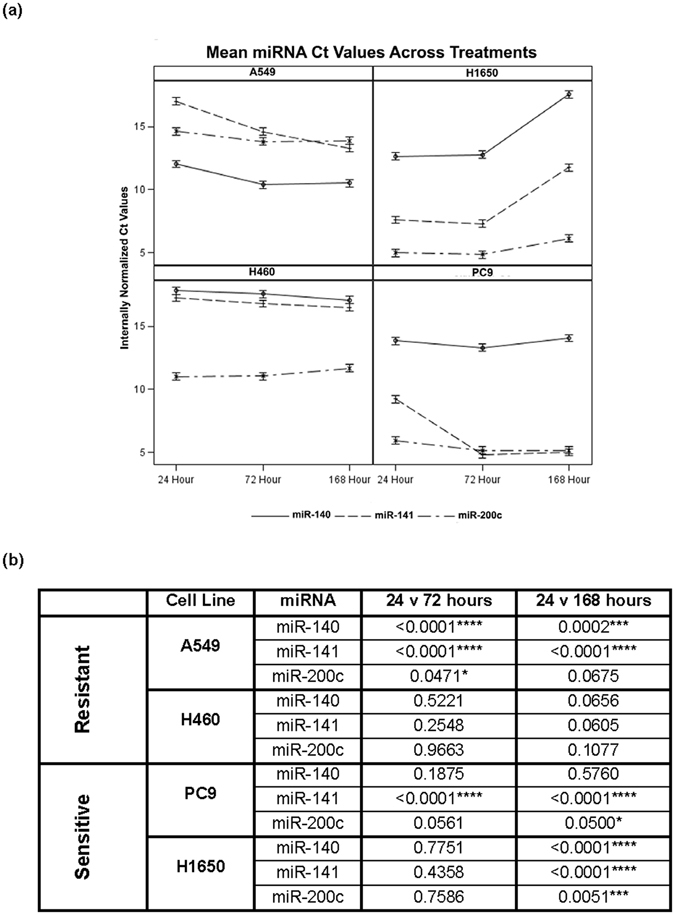
Time of TGFβ treatment reflects changes in endogenous miRNA gene expression. Changes in endogenous gene expression were analysed using a five-way ANOVA considering the variables: TGFβ treatment, SB-431542 treatment, time point, expression as internally normalized Ct values, and cell line, along with all interaction terms. (**a**) Data presented here is aggregated by averaging over treatments in order to capture overarching trends in miRNA and cell line patterns at multiple time points. Fine-scale trends were broken down by individual treatments as presented in Supplemental Figure 4. (**b**) Comparison of the significance of endogenous expression changes between time points samples and by individual miRNA genes in each cell line.

The miRNA expression trends did not differ significantly among treatment conditions, but differences across time points were observed (Supplementary Figure 4). An initial 2-way ANOVA comparing endogenous miRNA gene expression changes as internally-normalized Ct values within each cell line indicated that the most impactful variable governing endogenous expression change was the time of treatment. The 2-way ANOVA was not able to compare whether the expression changes correlated with other miRNA genes tested or the erlotinib- sensitivity status of a cell line. In order to capture this complexity, we used a 5-way ANOVA to identify significant interactions between five variables: 1) miRNA expression (Ct values), 2) time point sample was taken, 3) TGFβ treatment addition, 4) SB-431542 treatment addition, and 5) cell line. All combinations of factors were simultaneously calculated (5-way ANOVA Input in Supplementary Table 1, Ct averages in Supplementary File 1). The 5-way ANOVA revealed that treatments and miRNA expression levels are not related, and that the most influential experimental component was the time of treatment (Fig. 4a, Supplemental Figure 5 ). Supplemental Figure 5 shows that individual miRNA expression follow the same trends across treatments over time. For simplicity, since expression trends did not differ drastically between treatments, we chose to present the overarching miRNA expression trends generated as averages of treatments in each individual cell line at each time point (Fig. 4a). The table highlights the significance of endogenous expression changes among time points separated by miRNA gene in each cell line (Fig. 4b). Taken together, these data demonstrate that treatment was not impactful in the changes in endogenous miRNA expression, but the time of treatment was. Importantly, individual miRNA expression changes did not correlate with the erlotinib sensitivity of each cell line. From these data, we hypothesized that the impact of the time of treatment may be directly related to the cell cycle position of the cells.

### Time, not treatment, alters the cell cycle position of A549 and PC9 cells

To assess whether observed changes in miRNA gene expression correlate with cell cycle position, as a measure of time, A549 and PC9 cells were assessed for percentage of cells in each cell cycle position at each of the time points. Cells were treated and harvested as described for cell cycle analysis using propidium iodide staining and flow cytometry. For each sample, 10,000 events were counted to ensure percentages were not skewed by the differing number of cells present in each sample at the end of treatment. Overall proliferation following respective treatment times is shown in Supplementary Figures 6 and 7 as cell counts.

**Figure 5:**
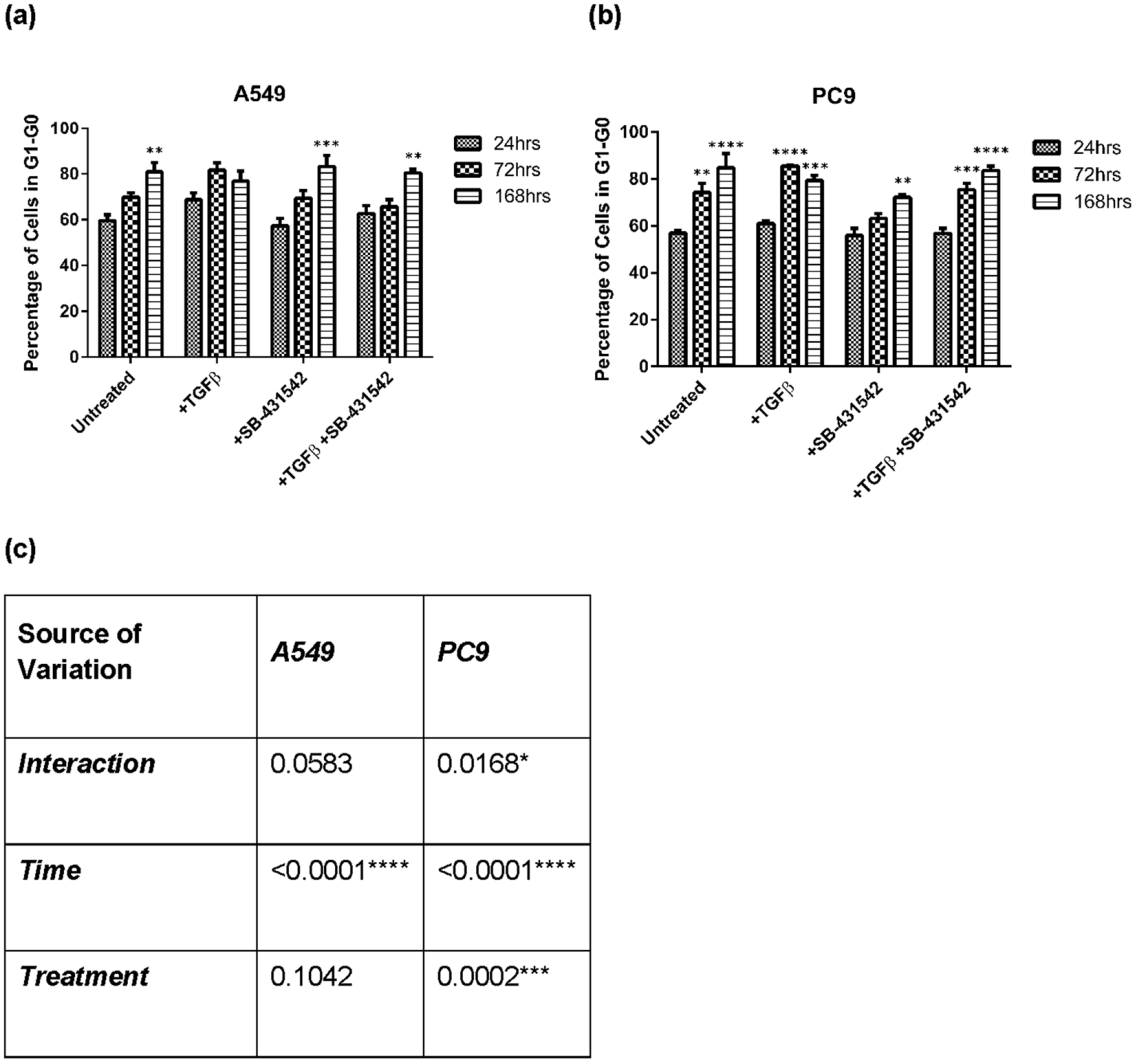
A549 and PC9 cells exit the cell cycle regardless of treatment with TGFβ or SB-431542. The graph reflects the percentage of (**a**) A549 or (**b**) PC9 cell populations in G_0_-G_1_ phase of the cell cycle at 24, 72, and 168 hours following treatment. Significance was determined using an unpaired t-test comparing the 72 and 168 hour time points individually to the 24 hour time point of the same treatment. (**c**) A two-way ANOVA was utilized to determine the significance of treatment and/or time point reflective of the percentage of cells in the G_0_-G_1_ phase of the cell cycle.

Irrespective of treatment, the percentage of A549 cells in the G_0_-G_1_ phase of the cell cycle increased over time of treatment. PC9 cells behaved similarly (Fig. 5). However, PC9 cells treated with TGFβ failed to continue to proliferate after 72 h while the percentage of cells in G_0_-G_1_ changed. To understand the impact of time and treatment on percentage of cells in the G_0_-G_1_ phase of the cell cycle, a 2-way ANOVA was performed within each individual cell line to capture the most impactful factor influencing the trends. The ANOVA confirmed that the most important factor governing the increasing number of cells in of G_0_-G_1_ phase was cumulative time of treatment. In A549 cells, time of treatment significantly explained cells in the G_0_-G_1_ phase of the cell cycle (p < 0.0001). In PC9, both treatment conditions (p < 0.0001), time of treatment (p = 0.0002), and the interaction of the two variables (p = 0.0168) had a significant impact on the percentage of cells in the G_1_-G_0_ phase of the cell cycle. Because cell cycle position interacted with time of treatment, we wondered whether a specific non- canonical signal transduction cascade downstream of TGFβ was activated that might impact cell cycle progression.

### TGFβ activation of non-canonical effectors ERK1/2 and AKT differs between A549 and PC9

**Figure 6:**
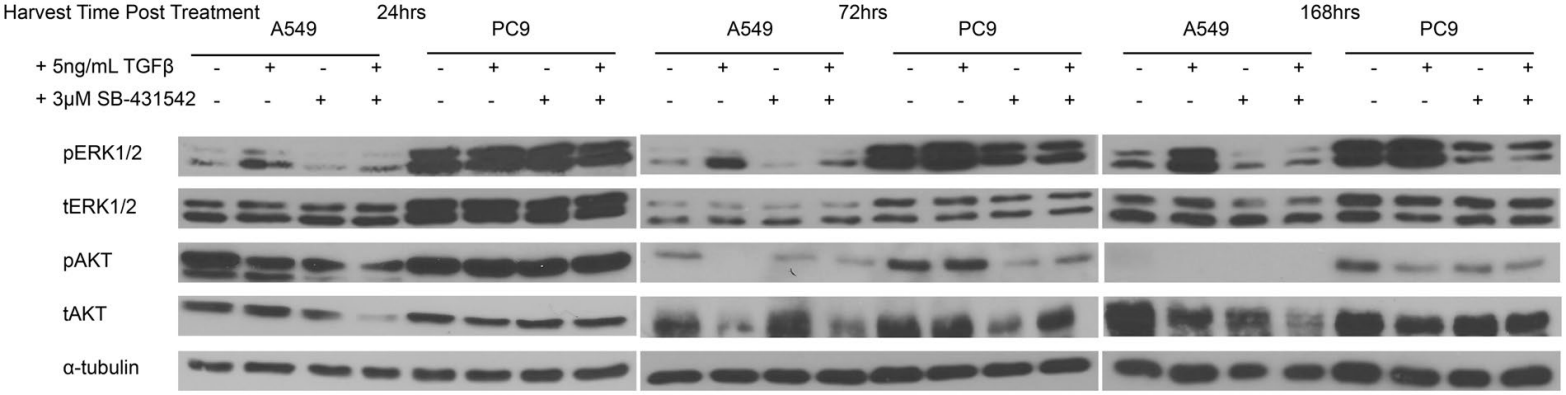
TGFβ modulation differentially impacts ERK and AKT activation between A549 and PC9. A549 and PC9 cells were plated, treated, and harvested as described in the methods. α-tubulin levels are representative of an individual lysate pool. Lysates profiled here are the same as in Fig. 2. ERK-MAPK and PI3K-AKT signaling are non-canonical signaling effectors of the TGFβ signaling pathway.

Since miRNA endogenous expression changes appeared to correlate with changes in the cell cycle rather than TGFβ treatment, we endeavoured to understand the impact of TGFβ treatment on non-canonical effectors known to drive growth and proliferation, Ras/MAPK and PI3K/AKT pathways. The same protein lysates profiled for the R-Smad effectors and EMT marker proteins in Figure 2 were assessed for both pERK1/2 and pAKT expression. Corresponding total protein expression of each across the same treatments and time points described above were measured by western blot (Fig. 6). In A549, pERK1/2 levels increase with TGFβ treatment across the time points while total protein levels remained constant. pAKT levels in A549 increased at the 24 hour time point but then diminish across time points while total levels of the protein were constant. In PC9, pERK1/2 and pAKT levels were elevated at the 24 hour time point, but both diminish over time without a decrease in total protein levels in the cells treated with SB-431542 with and without co-treatment with TGFβ. Densitometry performed on these blots can be seen in Supplemental Figure 8. These data suggest that the relationship between TGFβ and non-canonical growth and proliferation pathways and may explain why the changes in endogenous miRNA expression correlated with an increasing percentage of cells in the G_0_-G_1_ phase of the cell cycle.

## Discussion

In early stages of tumour development, TGFβ acts as a tumour suppressor preventing the proliferation, differentiation, and overall survival of the impacted cells. In later stages of tumour development, TGFβ shifts from tumour suppressive functions to promotion of tumorigenesis by driving the transcription of pro-EMT genes, which stimulate tumour cells to invade and metastasize^30, 31^. The role of TGFβ signaling in EMT is of particular interest to our group because the 13-gene miRNA signature not only stratified NSCLC into erlotinib-sensitive and erlotinib-resistant groups but was also able to discriminate between primary and metastatic tumours^6^, and multiple members of the miRNA signature have been shown to play either a promoting or repressing role in EMT in NSCLC^12, 32, 33^. For this reason, we endeavoured to understand the role of TGFβ signalling on the expression of microRNA genes dysregulated in erlotinib-sensitive compared with erlotinib–resistant cell lines.

TGFβ drives EMT by using the canonical signaling pathway, mediated by the R-Smads, which upregulate transcription responsible for the repression of epithelial characteristics^18^. Analysis of the TGFβ-driven R-Smad family members, showed a differential response to TGFβ treatment between the erlotinib-resistant, A549 cells, and erlotinib-sensitive, PC9 cells. Activated Smad2 and -3 expression was observed in both cell lines at similar levels at early time points of treatment. At the 168 hr time point, activated Smad2 levels return in A549 cells treated with TGFβ, compared to unchanging total Smad2, -3 and -4 levels across treatments. In PC9 cells after 168h of TGFβ treatment, the total expression of all TGFβ effectors tested was reduced suggesting the impact of some negative feedback mechanism. TGFβ is known for promoting EMT in late stages of tumour development, but in the early stages, it functions in an anti-EMT capacity^31^. We believe this cyclical pattern of TGFβ activation and R-Smad molecule repression to be indicative of TGFβ acting in an anti-EMT capacity in these cells.

To delve further into whether TGFβ treatment acted by different mechanisms between the two lines tested, we explored TGFβ-driven morphological changes and EMT marker protein expression changes. It is known that TGFβ treatment induces a very long, fibroblast-like phenotype in A549 cells (Supplemental Figure 2). Western blot analysis of the EMT markers E-cad, Vimentin, N-cad, and Zeb1 and immunofluorescence of E-cad and Vimentin shows that TGFβ treatment induced an expression phenotype consistent with EMT in A549 cells (Fig. 2b–d)^34^. However, this study is the first to demonstrate biological differences in “epithelial” NSCLC cell lines, like PC9 cells, treated with TGFβ. In PC9 cells, the morphology after TGFβ treatment is unchanged. Interestingly, PC9 cells treated with the TGFβ inhibitor, SB-431542, with and without co-stimulation with TGFβ develop a morphology consistent with an EMT- intermediate phenotype known as “metastable” suggesting that the inhibition of TGFβ in PC9 cells may play a role in the induction of EMT (Supplementary Figure 2)^29, 35^. This observation, as well as that of the change in expression of the R-Smads in these cells, is consistent with the TGFβ-paradox theory and also correlates with the signature’s ability to stratify primary and metastatic lesions. To test whether TGFβ inhibition induced EMT initiation in PC9 cells, we profiled EMT protein markers to determine if the morphological change was indeed indicative of an EMT intermediate. While PC9 cells treated with the TGFβ inhibitor, SB-431542, undergo a morphological change consistent with EMT initiation, the western blot and immunofluorescence analyses revealed that the cadherin switch, that is essential for full-EMT, did not occur in response to treatment^36^. Taken together, these data suggest that while TGFβ may act as a pro-tumorigenic, pro-EMT fashion in A549 cells, it may play an anti-EMT and protective role in PC9 cells because the inhibition of TGFβ did not induce a complete EMT transition in these cells.

Since A549 and PC9 cells appeared to represent either side of the TGFβ paradox, we sought to elucidate whether TGFβ directly regulated the expression of the candidate signature miRNA genes to understand whether the differing impact of TGFβ observed by R-Smad and EMT marker expression was also differentially regulating the expression of some of the signature miRNA genes. We expected TGFβ to directly regulation the expression of the signature miRNA and from there we expected to be able to triangulate a relationship between erlotinib- sensitivity, TGFβ signaling, and the 13-miRNA signature to determine therapeutically-relevant, secondary targets for overcoming inherent or acquired erlotinib-resistance. To test if TGFβ was directly influencing the expression of miR-200c and -141, we performed a ChIP assay to determine whether TGFβ induced the binding of Smad4 to an SBE site in the shared promoter of mir-200c/-141. These two miRNA genes have very different baseline expression profiles between the mesenchymal, A549, and epithelial, PC9, cell lines. We showed that TGFβ treatment induced Smad4 interaction with the shared mir-141/-200c promoter only in PC9 cells. However, in PC9 cells endogenous miR-141 and -200c expression at 24 hours after treatment showed no impact of any treatment condition, suggesting that TGFβ signaling may not be important in this context. Importantly, Smad4 must be bound to activated Smad2 or -3 to carry out transcriptional control, and we did not test whether pSmad2/3 was present with Smad4.

While we did not observe a change in endogenous expression of any of the three miRNA genes in response to treatment, we did observe that the change in expression of miR-200c and -141 in response to changes time of treatment, and we believe that time is reflective of cell cycle position. Importantly, miR-200c and −141 are thought to be under coordinated transcriptional regulation because of an overlapping promoter region^37^. Our data suggests that, at least in these treatment conditions and cell lines tested, miR-141 and -200c are not commonly regulated as is expected of genes that share a promoter region. We also observed that the trends in expression changes did not segregate the two erlotinib-resistant lines, A549 and H460 cells, from the two erlotinib-sensitive lines, PC9 and H1650 cells, suggesting that changes in the expression of these miRNA did not correlate with erlotinib-resistance or EMT status (Fig. 4/Supplemental Figure 5).

Using a 5-way ANOVA, we discovered that that the most important factor governing the changes in endogenous miRNA expression was the time of treatment. Thus, we investigated whether cell cycle stage could impact the expression of these genes. In Supplemental Figure 9, we interrogated the putative transcription factor binding sites of one cell cycle regulated effector, ELK1, using the ChipMAPPER algorithm^21, 22^. The analysis revealed putative ELK1 sites in the promoters of 12 out of 13 of the miRNA genes profiled, supporting our hypothesis that cell cycle progression may control the expression of the candidate miRNA genes. Analysis of the cell cycle position of A549 and PC9 cells across the same treatments and time points revealed that as time of treatment increased, the percentage of cells in the G_1_-G_0_ phase of the cell cycle increased, except in TGFβ treated cells at the final time point (Fig. 5). Importantly, the impact of treatment alone on cell cycle stage was only significant in PC9 cells (Fig. 5). Supplemental Figures 6 and 7 illustrates cell counts, reflective of doublings, in both 1% and 10% serum across treatment conditions. PC9 cells failed to continue to growth in the presence of TGFβ and 1% serum which may explain the reduction of cells in G_1_-G_0_ phase of the cell cycle at 168 h. Further experimentation will be necessary to understand this modest but significant decline.

Finally, because of the observation that cell cycle position may be important in expression of the miRNA examined in this study, we interrogated the activation of TGFβ non-canonical growth and proliferation pathways, Ras/MAPK and PI3K/AKT, to determine if they may play a role in the relationship of cell cycle position and endogenous miRNA expression. pERK activation increased across the time points in A549 cells, and its activation may influence the re-emergence of pSmad2 levels at 168 hours because pERK is known to phosphorylate the linker region of Smad2 to stabilize the signal^18^. pERK signalling is also required for TGFβ- driven EMT, consistent with the increase in pERK signal in A549 cells undergoing TGFβ-induced EMT^29^. PC9 cells harbour an EGFR-activating mutation resulting in the constant expression of pERK and pAKT. Perhaps most interestingly, treatment with the TGFβ receptor inhibitor, SB-431542, resulted in the reduction of both signals regardless of co-treatment with TGFβ ligand. SB-431542 is a competitive ATP binding site kinase inhibitor and has been shown to disallow ERK, JNK, or p38 pathway activation from other signals or their response to serum^38^. These data suggest that, at least in PC9 cells, the perpetual activation of ERK and AKT signals from active EGFR signaling may rely on basal activation from TGFβRII in order to persist. We anticipate testing this using a TGFβ-receptor knock-down to observe whether the same impact on ERK and AKT signals is observed.

Our future experiments will attempt to fill the gaps noted from this work. We will determine whether the remaining erlotinib-sensitive cell lines used to generate gene expression data have a similar response to long term TGFβ treatment even though we know that erlotinib-sensitive tumours also have metastatic capability. We will also determine if erlotinib response is altered by time in treatment as miRNA expression and cell cycle position were. We will test whether the expression of ELK1 in cells is important for cell cycle progression in this context because the shared promoter of mir-141 and -200c contains an ELK1 binding site. We might also determine if E2F sites are present and active because TGFβ-driven, DNA-binding Smad complexes have been shown to interact with cell cycle regulating elements^39, 40^. Therefore, it is possible that Smad4 binding to the SBE in PC9 cells does requires coordinate cell cycle regulation, through ELK1, to regulate the expression of miR-141. The presence of known cell cycle responsive elements in the promoters of most of the 13-signature miRNA suggests that the cell cycle may play a role in governing the expression levels of these miRNA genes. Understanding the mechanism of regulation of the signature miRNA genes might help us further understand whether TGFβ signalling is a driver of EMT and metastasis or a passenger alongside cell cycle-dependent regulation of these genes.

Our original hypothesis that TGFβ directly regulated the expression of the microRNA gene signature and that it modulated gene expression differently in erlotinib-resistant versus erlotinib-sensitive cells was founded on a bioinformatics analysis of these genes with little regard for cellular context. We found that TGFβ is likely not directly responsible for control of the expression of the microRNA genes we tested. However, we still find it an attractive therapeutic target if we can understand the cellular or tumoural context wherein targeting this cytokine impacts NSCLC patient survival.

## Methods

### Cell Culture, Protein harvest, Immunofluorescence, and Western Blot

A549, PC9, H460, and H1650 cell lines (NSCLC) were purchased from ATCC. They were cultured in RPMI 1640 supplemented with 10% FBS and maintained in a humidified incubator at 37 °C at 5% CO^2^. Cells were seeded in 6 well plates and were allowed to grow under maintenance media conditions for 48 hours prior to treatments. Cells undergoing 24 hours of treatment were plated 4 × 10^4^ cells/well, and 72 and 168 hour treated samples were plated at 1 × 10^4^ cells. Cells were treated with SB-431542 (3 µM) and/or TGFβ (5 ng/ml) under minimal serum (1%) conditions for time frames specified. If treatment times exceeded 72 hours, treatment media was replenished at the 72-hour time point. Whole-cell extracts were collected using RIPA buffer (50 mM Tris- HCl, 1% NP-40, 150 mM NaCl, 1 mM EDTA, 0.25% DOC, 10^%^ Glycerol, in ddH_2_O) and protein content was quantified using a BCA kit (Thermo Fisher) prior to western blotting. Proteins were separated using SDS-PAGE and were transferred to a nitrocellulose membrane. Expression and/or activation of specific proteins (pSmad2, tSmad2, pSmad3, tSmad3, tSmad4, α-tubulin, pERK1/2, tERK1/2, pAKT, tAKT, E-cad, Vimentin, N-cad, and Zeb1) was assess by western analysis using antibodies purchased from Cell Signaling Technology. Immunofluorescence was performed using Alexa Fluor-conjugated antibodies of the specific clone of E-cadherin and Vimentin used for western blotting (Cell Signaling Technology). Immunofluorescence was measured using the AMG EVOS microscope with built-in EVOS software (Thermo Fisher). Cell morphology images was recorded using the Zeiss AxioObserver Microscope and processed using the AxioVision software.

### Chromatin Immunoprecipitation (ChIP)

ChIP assays were carried out with the Simple ChIP Plus Enzymatic Chromatin IP Kit (Cell Signaling Technology) to measure Smad4 binding to two putative SBE sites in the shared promoter of miRNA-141 and -200c at -1645/-1247 and -1793/-1395 from each transcriptional start site respectively. Cells were plated at 5 × 10^5^ cells per dish in 10 cm dishes for 48 hours prior to a media change to 1% FBS-containing RPMI +/− 5ng/ml TGFβ1 treatment for 24 hours. After treatment, cells were cross-linked, processed, and digested as described in the Simple ChIP protocol (Cell Signaling Technology). Samples were divided following digestion and chromatin complexes were immunoprecipitated with Smad4 antibody (20 µl/ChIP) against a non-specific rabbit IgG (1 µl/ChIP) overnight and then pulled down with magnetic ChIP-grade protein G beads for 2 hours (Cell Signalling). Immunoprecipitated samples were washed, uncrosslinked, and DNA was prepared as described in the Simple ChIP protocol (Cell Signalling). SYBR Green qRT-PCRs (Applied Biosystems) were performed with negative-control α-Satellite and positive-control ID1 Smad4-specific control primers against the experimental region containing the two putative SBEs in the shared promoter of miR-141/-200c (Forward: GCATTACTCAGCAAATCCTTAC; Reverse: CCCGACAGGTGATTGCC. Primers designed in-house and produced by IDT). Data was analysed using the Percent Input method where signals from ChIP samples are represented as a percentage of the total chromatin input. Each individual experiment was replicated in triplicate for each primer set and processed using the 2% input method described in the Cell Signaling Technology protocol. Data represented is for three biological replicates (n = 3). P-values were generated using paired t-tests comparing each TGFβ treated sample to its respective untreated sample.

### Real-time PCR analysis of miRNA expression

Total small RNA was harvested from the cells using the mirVANA™ miRNA isolation kit (Life Technologies). cDNA was synthesized for U6, miR-140, miR-141, and miR-200c using the TaqMan MicroRNA Reverse Transcription kit and corresponding reverse transcription TaqMan primers for U6, miR-140, miR-141, and miR- 200c (Life Technologies). cDNA was then subjected to quantitative Real Time PCR (qRT-PCR) using TaqMan Mastermix II with no UNG, and corresponding TaqMan microRNA assay primers (Life Technologies). qRT-PCR were performed by a 7900HT Fast Real-Time PCR system (ABI) and all reactions were run in duplicate with corresponding positive and negative controls. The data was analysed using a 5-way ANOVA following internal normalization of raw Ct values to the internal U6 as the normalization probe.

### Propidium Iodide (PI) and Flow Cytometry

A549 and PC9 cells were subjected to the same treatments and time points as previously described. Specifically, cells were rinsed in PBS at point of harvest, trypsinised, and collected in a 15 ml conical tube. Cells are centrifuged at 1500 rpm and the supernatant is removed. Cells are washed once with cold PBS, pelleted, and the supernatant removed. Finally, the cell pellet was resuspended in 400 µl of cold PBS and then 1 ml of cold, 100%, molecular biology grade ethanol was added to each sample dropwise while gently vortexing and then samples were placed on ice for 30 minutes. Cells were pelleted by centrifugation and the supernatant removed, and then washed in cold PBS/1%BSA. The pellet was resuspended in 0.3 ml of PI solution (1X PBS/1% BSA/50 µg/ml PI/0.5 mg/ml RNase A). Samples were incubated in the PI solution for at least 30 minutes at 4 °C protected from light. Samples were assayed on the Attune Flow Cytometer acoustic focusing cytometer, and 10,000 cells from each sample were profiled for PI emission, and data was collected with the Attune-specific software provided (Applied Biosystems/Thermo Fisher). Percentage of total cells in each phase of the cell cycle was determined using the cell cycle analysis platform in the FlowJo V10 software (FlowJo).

### Statistics

To analyse changes in endogenous gene expression data generated by qRT-PCR described above, a five-way ANOVA was performed using the following variables: treatment with TGFβ, treatment with SB-431542, time point, expression as internally normalized Ct values, and cell line, along with all interaction terms. The overall F-test, followed by partial F-tests were used to determine significant effects. Following the ANOVA, post- hoc comparisons were made for significant terms in the ANOVA using two-sample *t*-tests to compare subgroups of interest. Tests were determined to be significant if p-values were less than 0.05. All analyses were performed in SAS Version 9.3 or above (SAS Institute Inc., Cary, NC).

## Electronic supplementary material

Supplementary Figures

Supplementary Data Set 1
