# Supplementary material for: A microRNA signature of response to erlotinib is descriptive of TGFβ behaviour in NSCLC
Source: Sci Rep. 2017 Jun 23;7:4202. doi: 10.1038/s41598-017-04097-7 (PMC5482799; doi:10.1038/s41598-017-04097-7)
Supplement: Supplementary file 1 — Supplementary Figures [file 41598_2017_4097_MOESM1_ESM.doc]

**A microRNA signature of response to erlotinib is descriptive of TGFβ behaviour in**

**NSCLC**

**Madeline Krentz Gober, James P. Collard, Katherine Thompson and Esther P. Black**

**Supplementary Figure 1:**


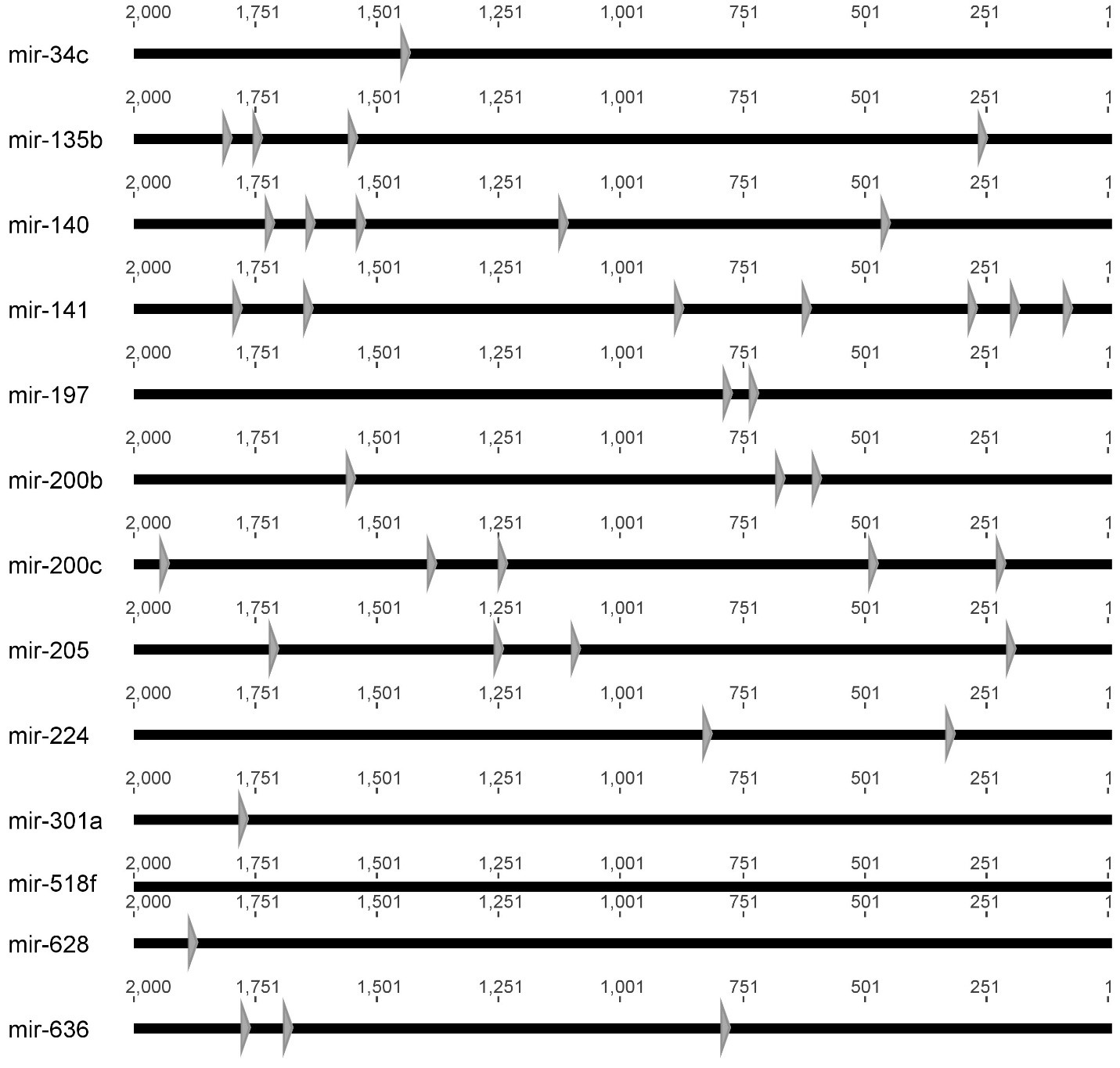


***Supplementary Figure 1: Signature microRNA genes contain SBE elements.*** Promoter analysis was conducted using the ChipMAPPER algorithm [1]. Twelve out of 13 of the signature microRNA genes contain putative SBE elements as represented by the triangle with conservative E-values less than or equal to 25 and a score greater than 3.0.

**Supplementary Figure 2**

**A**


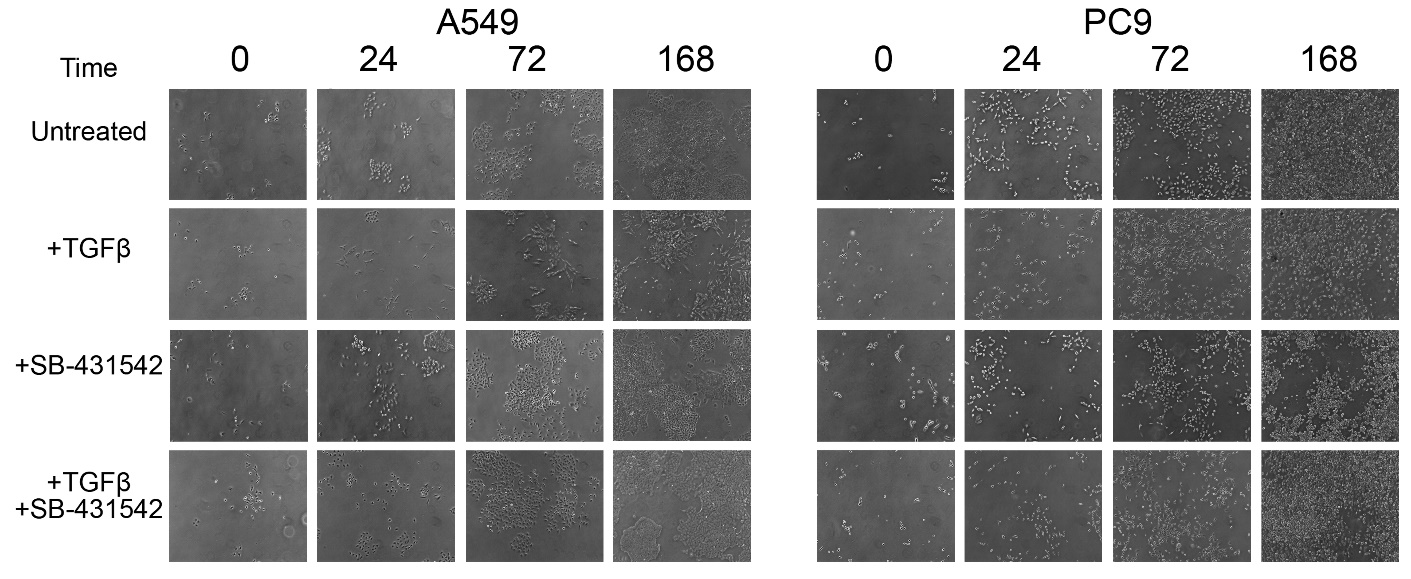


**B**


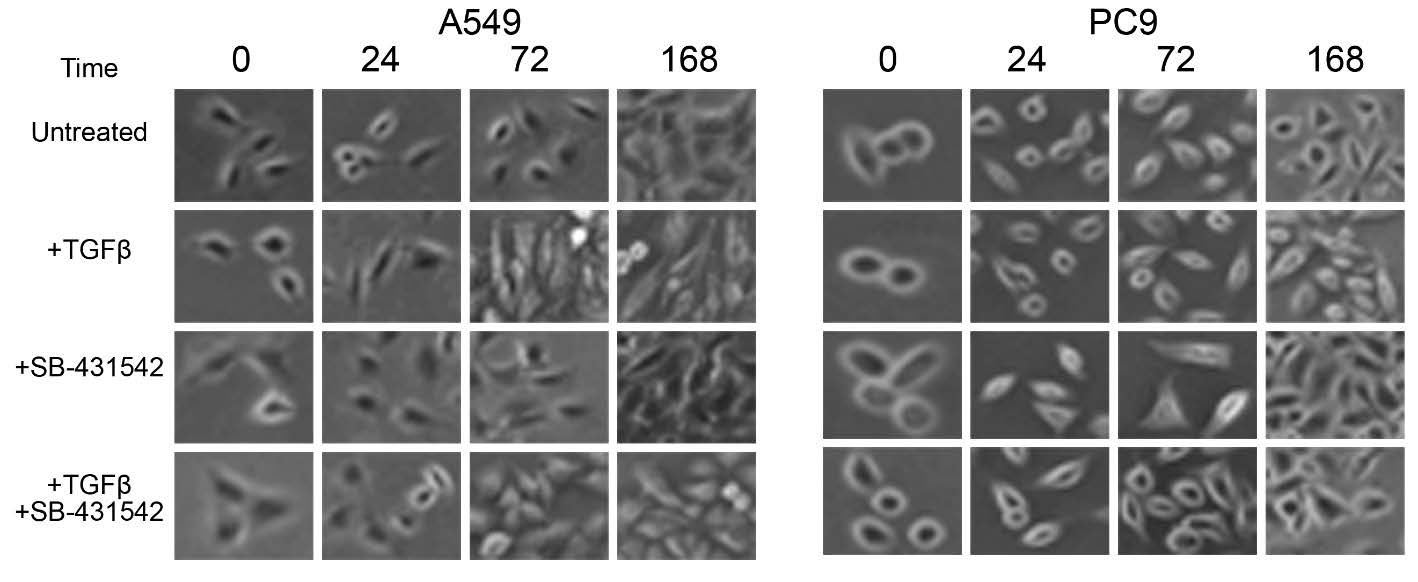


**Supplementary Figure 2: TGFβ induces a mesenchymal phenotype in A549, but inhibition generates an EMT-intermediate phenotype in erlotinib-sensitive, PC9 cells.** A549 and PC9 cells were treated as described in the methods. Bright field images of cell morphology were acquired using the microscope and software described in the methods. **(a)** Shows full-sized bright-field images taken at 5X magnification, and **(b)** shows a closer representation of the morphology changes.

**Supplementary Figure 3**

**A B**


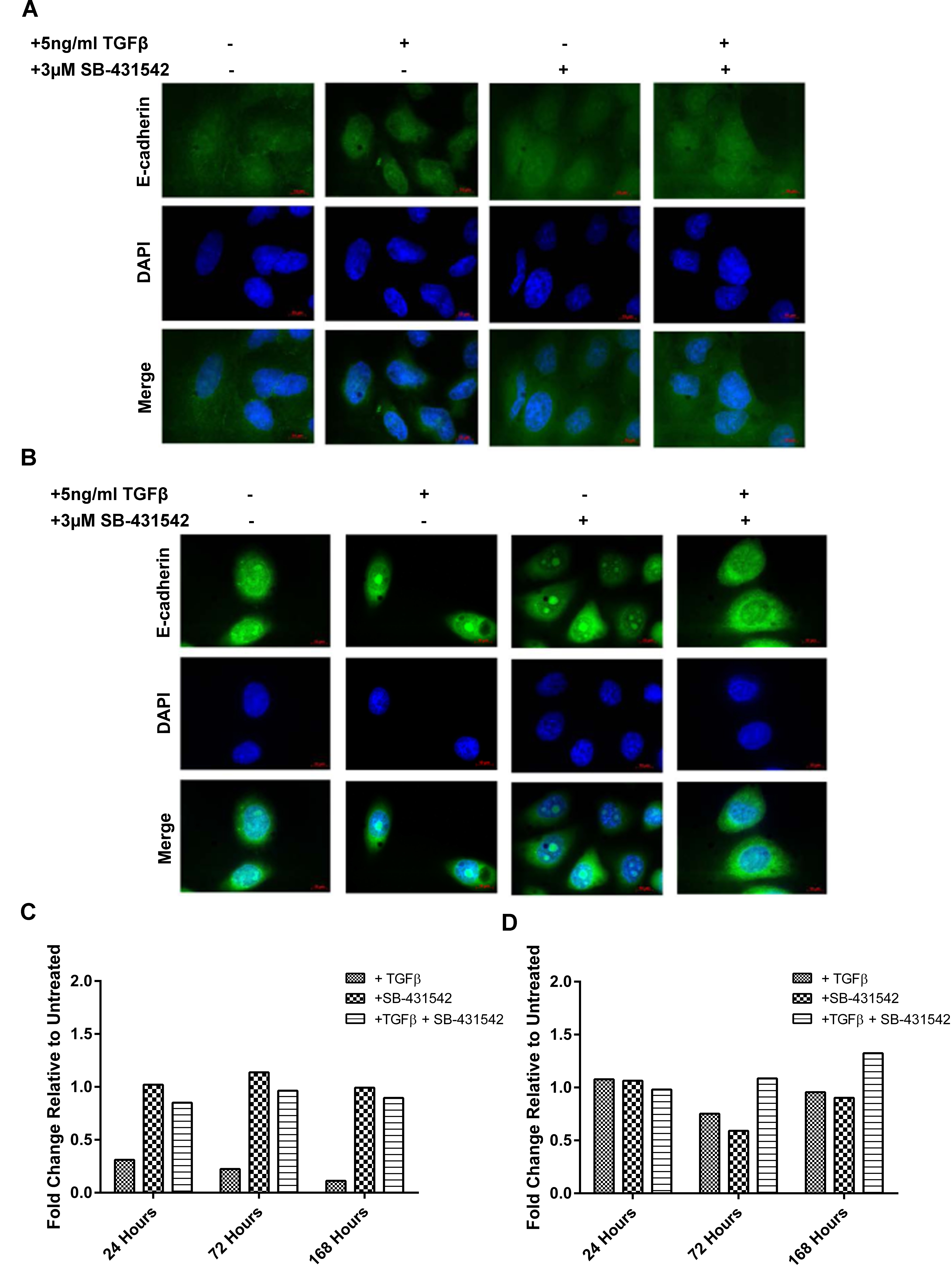


**Supplementary Figure 3: E-cad expression in response to treatment. (A)** E-cad mRNA levels quantified by qRT-PCR in A549 (n=1). **(B)** E-cad mRNA levels quantified by qRT-PCR in PC9 (n=1). mRNA levels are demonstrated as fold change relative to respective untreated samples which are standardized to a fold change of 1.

**Supplementary Figure 4**


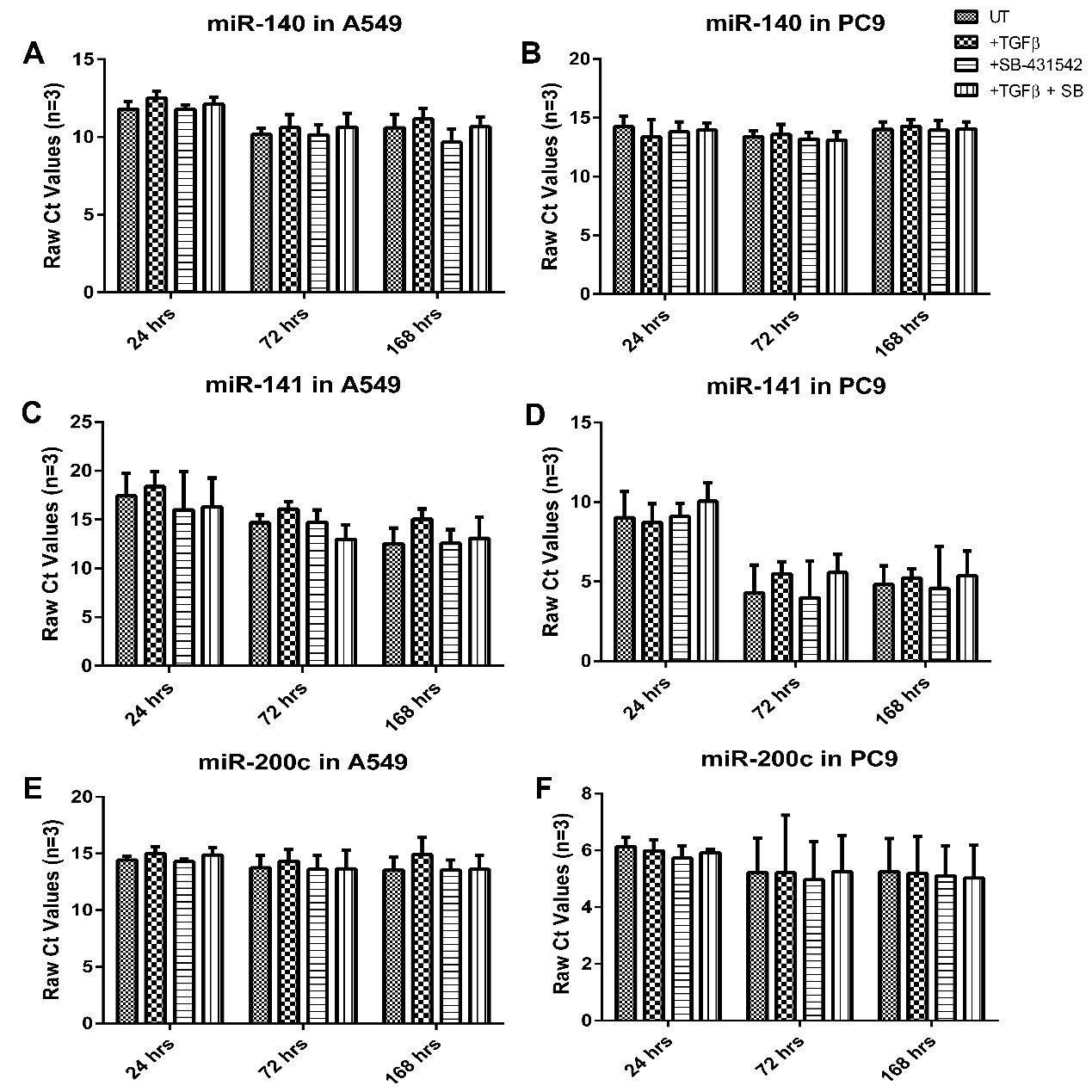


***Supplementary Figure 4: Normalized Ct values demonstrate that time change, not individual treatment, affects endogenous miRNA expression changes in A549 and PC9 cells. (A-E)*** *Raw miRNA expression levels using* qRT-PCR experiments as described in Figure 4 and Supplemental Figure 5.

**Supplementary Table 1**

**A**

|  | **Class Level Information** | |
| --- | --- | --- |
| **Class** | **Levels** | **Values** |
| **Time** | 3 | 168 Hour 24 Hour 72  Hour |
| **expr** | 3 | miR-140 miR-141 miR200 |
| **status** | 4 | A549 H1650 H460 PC9 |
| **TGF** | 2 | N Y |
| **SB** | 2 | N Y |

**B**

| **Source** | **DF** | **Sum of**  **Squares** | **Mean Square** | **F Value** | **Pr > F** |
| --- | --- | --- | --- | --- | --- |
| **Model** | 14  3 | 8088.98443  2 | 56.566325 | 57.40 | <.0001 |
| **Error** | 28  8 | 283.797472 | 0.985408 |  |  |
| **Corrected Total** | 43  1 | 8372.78190  4 |  |  |  |

**C**

| **Source** | **DF** | **Type III SS** | **Mean Square** | **F Value** | **Pr > F** |
| --- | --- | --- | --- | --- | --- |
| **Time** | 2 | 79.952136 | 39.976068 | 40.57 | <.0001 |
| **Expr** | 2 | 1885.22176  9 | 942.610885 | 956.57 | <.0001 |
| **Time*expr** | 4 | 54.519027 | 13.629757 | 13.83 | <.0001 |
| **TGF** | 1 | 10.424204 | 10.424204 | 10.58 | 0.0013 |
| **Time*TGF** | 2 | 1.076126 | 0.538063 | 0.55 | 0.5798 |
| **expr*TGF** | 2 | 1.079149 | 0.539574 | 0.55 | 0.5790 |
| **Time*expr*TGF** | 4 | 0.646410 | 0.161602 | 0.16 | 0.9565 |
| **Source** | **DF** | **Type III SS** | **Mean Square** | **F Value** | **Pr > F** |
| **SB** | 1 | 1.546384 | 1.546384 | 1.57 | 0.2113 |
| **Time*SB** | 2 | 0.471393 | 0.235697 | 0.24 | 0.7874 |
| **expr*SB** | 2 | 1.520563 | 0.760281 | 0.77 | 0.4633 |
| **Time*expr*SB** | 4 | 0.324106 | 0.081027 | 0.08 | 0.9878 |
| **TGF*SB** | 1 | 0.134397 | 0.134397 | 0.14 | 0.7122 |
| **Time*TGF*SB** | 2 | 0.541967 | 0.270983 | 0.27 | 0.7598 |
| **expr*TGF*SB** | 2 | 1.295853 | 0.647926 | 0.66 | 0.5189 |
| **Time*expr*TGF*SB** | 4 | 0.965323 | 0.241331 | 0.24 | 0.9126 |
| **Status** | 3 | 3240.47954  1 | 1080.159847 | 1096.16 | <.0001 |
| **Time*status** | 6 | 364.036762 | 60.672794 | 61.57 | <.0001 |
| **expr*status** | 6 | 2266.03494  2 | 377.672490 | 383.27 | <.0001 |
| **Time*expr*status** | 12 | 127.856402 | 10.654700 | 10.81 | <.0001 |
| **status*TGF** | 3 | 2.810260 | 0.936753 | 0.95 | 0.4165 |
| **Time*status*TGF** | 6 | 4.252351 | 0.708725 | 0.72 | 0.6344 |
| **expr*status*TGF** | 6 | 2.875868 | 0.479311 | 0.49 | 0.8183 |
| **Time*expr*status*TGF** | 12 | 2.785062 | 0.232089 | 0.24 | 0.9964 |
| **status*SB** | 3 | 12.852156 | 4.284052 | 4.35 | 0.0051 |
| **Time*status*SB** | 6 | 0.954675 | 0.159113 | 0.16 | 0.9866 |
| **expr*status*SB** | 6 | 6.327867 | 1.054644 | 1.07 | 0.3804 |
| **Time*expr*status*SB** | 12 | 3.120940 | 0.260078 | 0.26 | 0.9939 |
| **status*TGF*SB** | 3 | 6.390615 | 2.130205 | 2.16 | 0.0927 |
| **Time*status*TGF*SB** | 6 | 2.107473 | 0.351246 | 0.36 | 0.9058 |
| **expr*status*TGF*SB** | 6 | 4.105405 | 0.684234 | 0.69 | 0.6544 |
| **Tim*expr*stat*TGF*SB** | 12 | 2.275306 | 0.189609 | 0.19 | 0.9987 |

**Supplementary Table 1: Output of 5-way ANOVA analysis. (Data pairs with Supplementary figures 4 and 5) (A)** Variable names used in analysis. **(B)** Five-way ANOVA Overall F-test of the endogenous miRNA data. **(C)** Tests of the effects within the five-way ANOVA.

**Supplementary Figure 5**

**
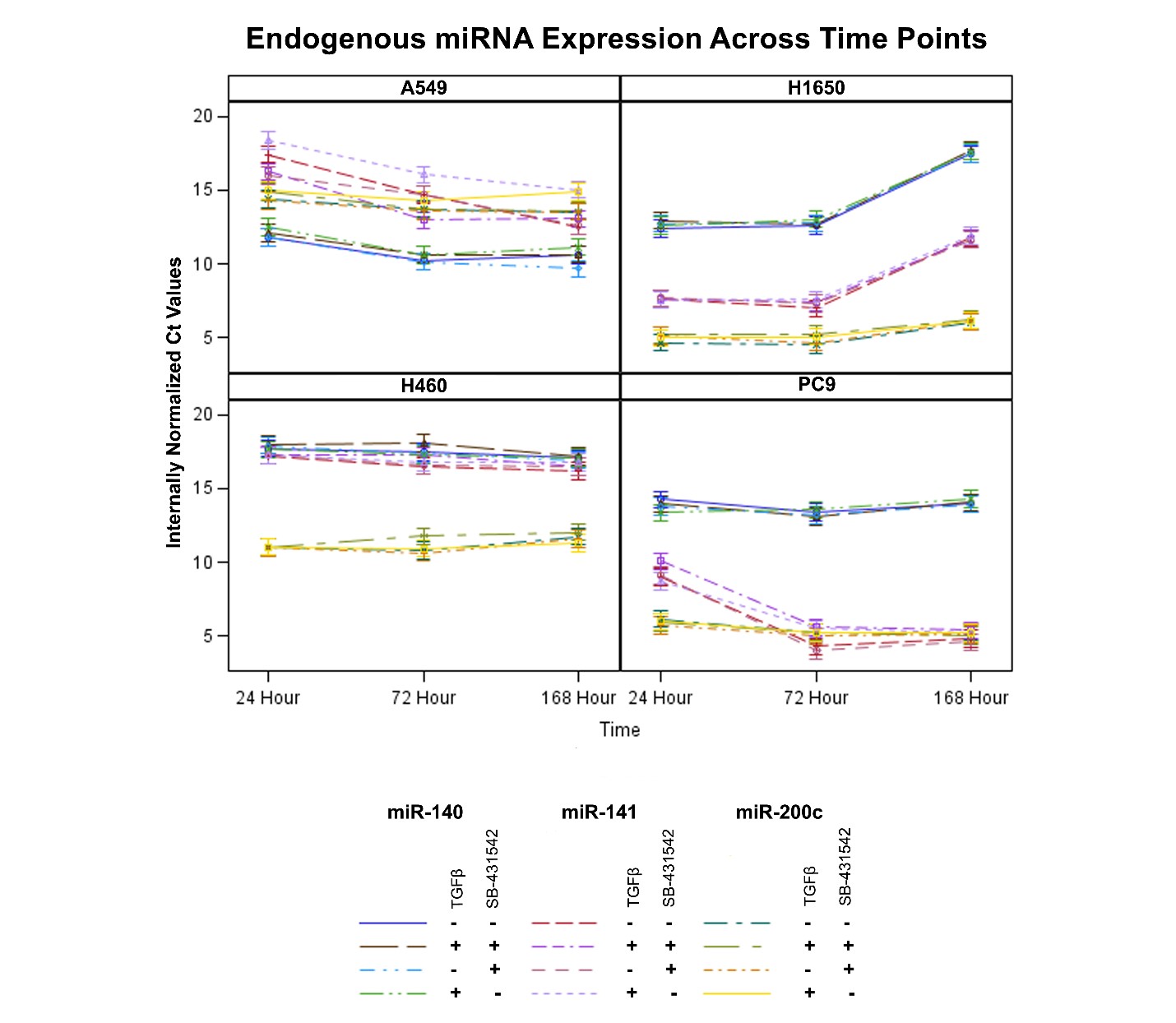
**

**Supplementary Figure 5: *Time of treatment has the most significant influence on miRNA expression changes.*** Changes in endogenous gene expression were analyzed using a five-way ANOVA considering the variables: TGFβ treatment, SB-431542 treatment, time point, expression as internally normalized Ct values, and cell line, along with all interaction terms.

**Supplementary Figure 6**

**
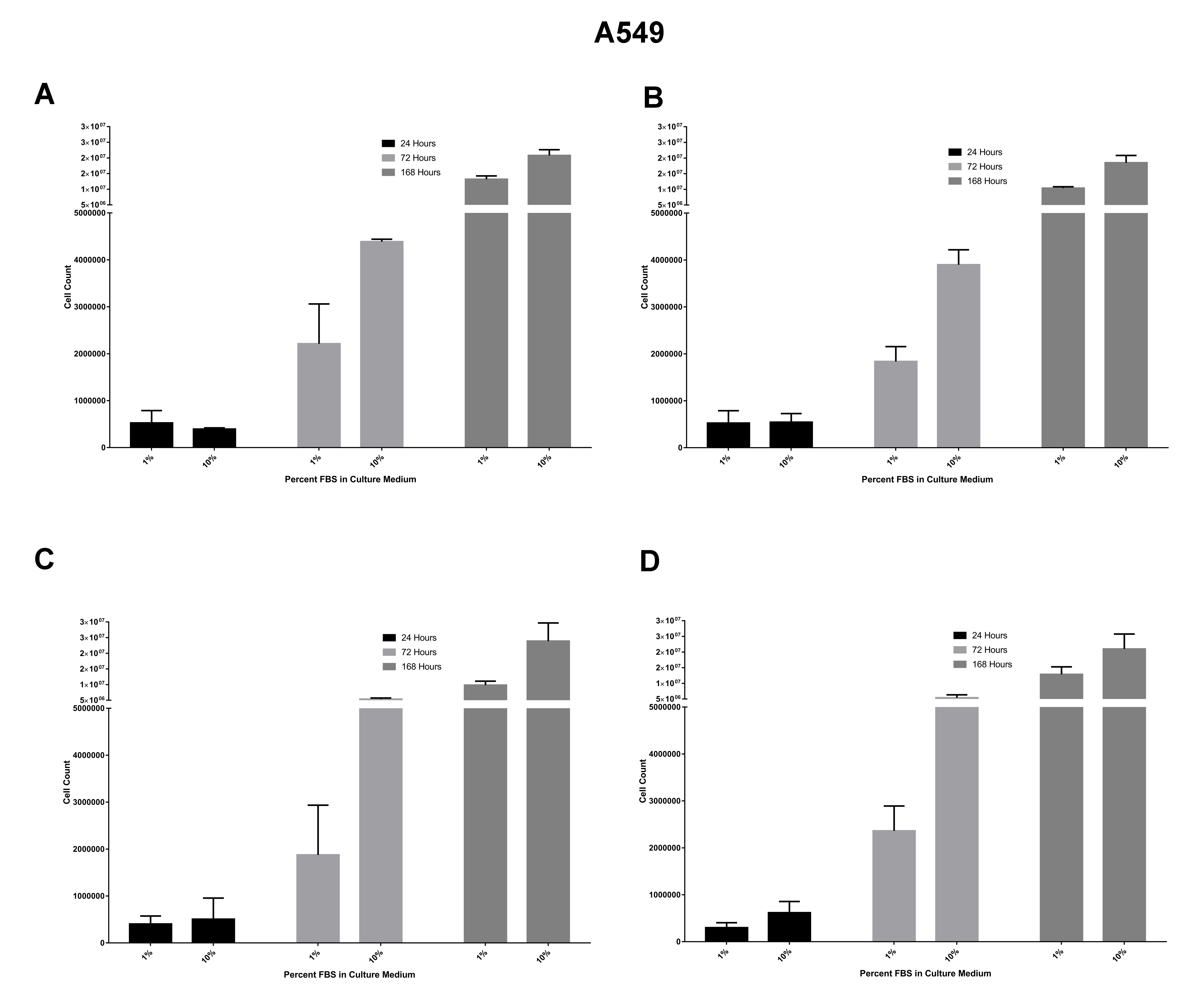
**

***Supplementary Figure 6: A549 cell counts following corresponding treatments and time points comparing growth in 1% and 10% serum media.* (A)** Untreated **(B)** +5ng/ml TGFβ **(C)** +3µM SB-431542 **(D)** +5ng/ml TGFβ +3µM SB-431542. Cells were plated at 1 x 10^4^ cells/well in a 6-well dish 48 hours prior to 0 hour treatment introduction and counted at the appropriate harvest time using a hemocytometer. (n=2)

**Supplementary Figure 7**


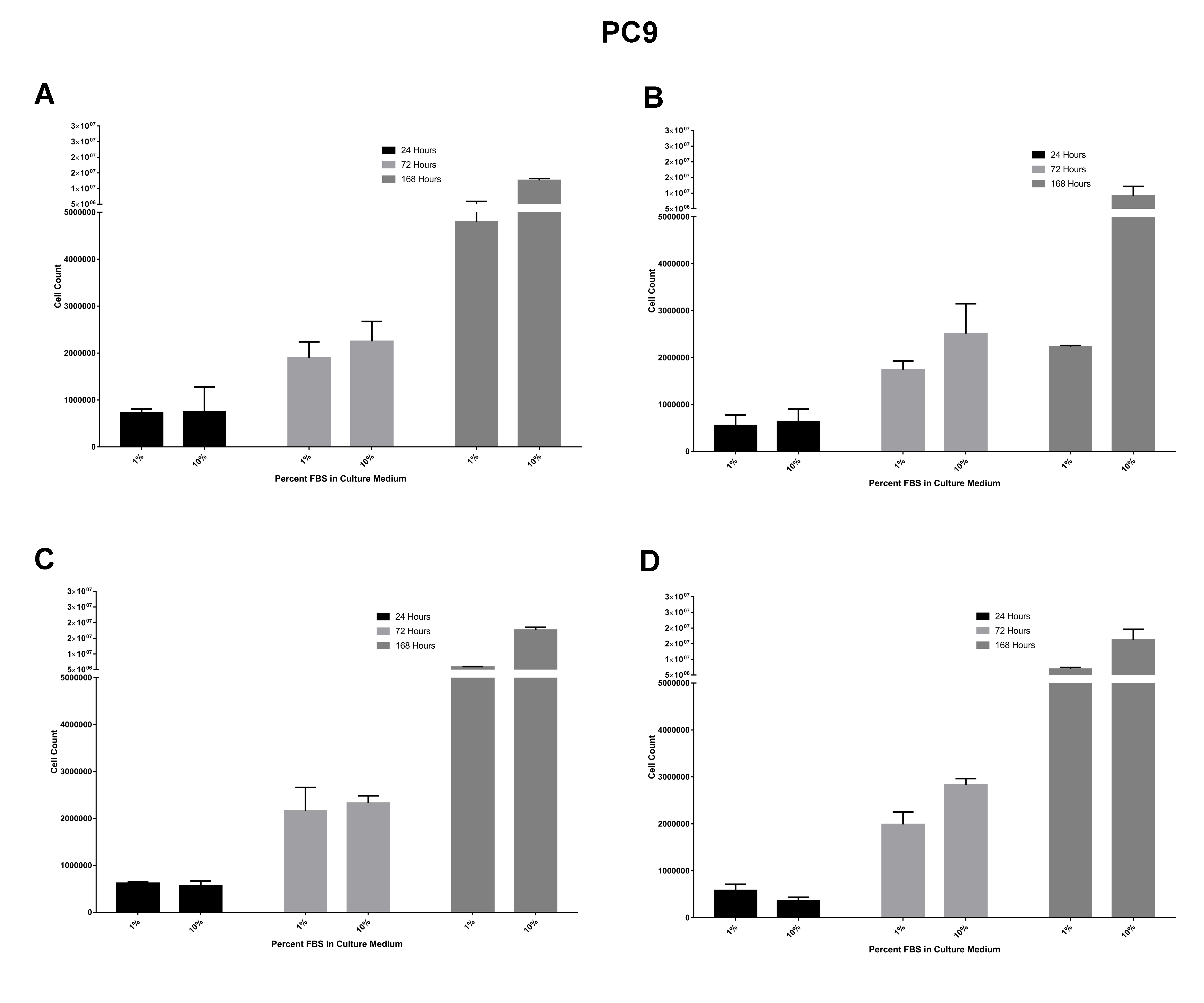


***Supplementary Figure 7: PC9 cell counts following corresponding treatments and time points comparing growth in 1% and 10% serum media.* (A)** Untreated **(B)** +5ng/ml TGFβ **(C)** +3µM SB-431542 **(D)** +5ng/ml TGFβ +3µM SB-431542. Cells were plated at 1 x 10^4^ cells/well in a 6-well dish 48 hours prior to 0 hour treatment introduction and counted at the appropriate harvest time using a hemocytometer. (n=2)

**Supplementary Figure 8
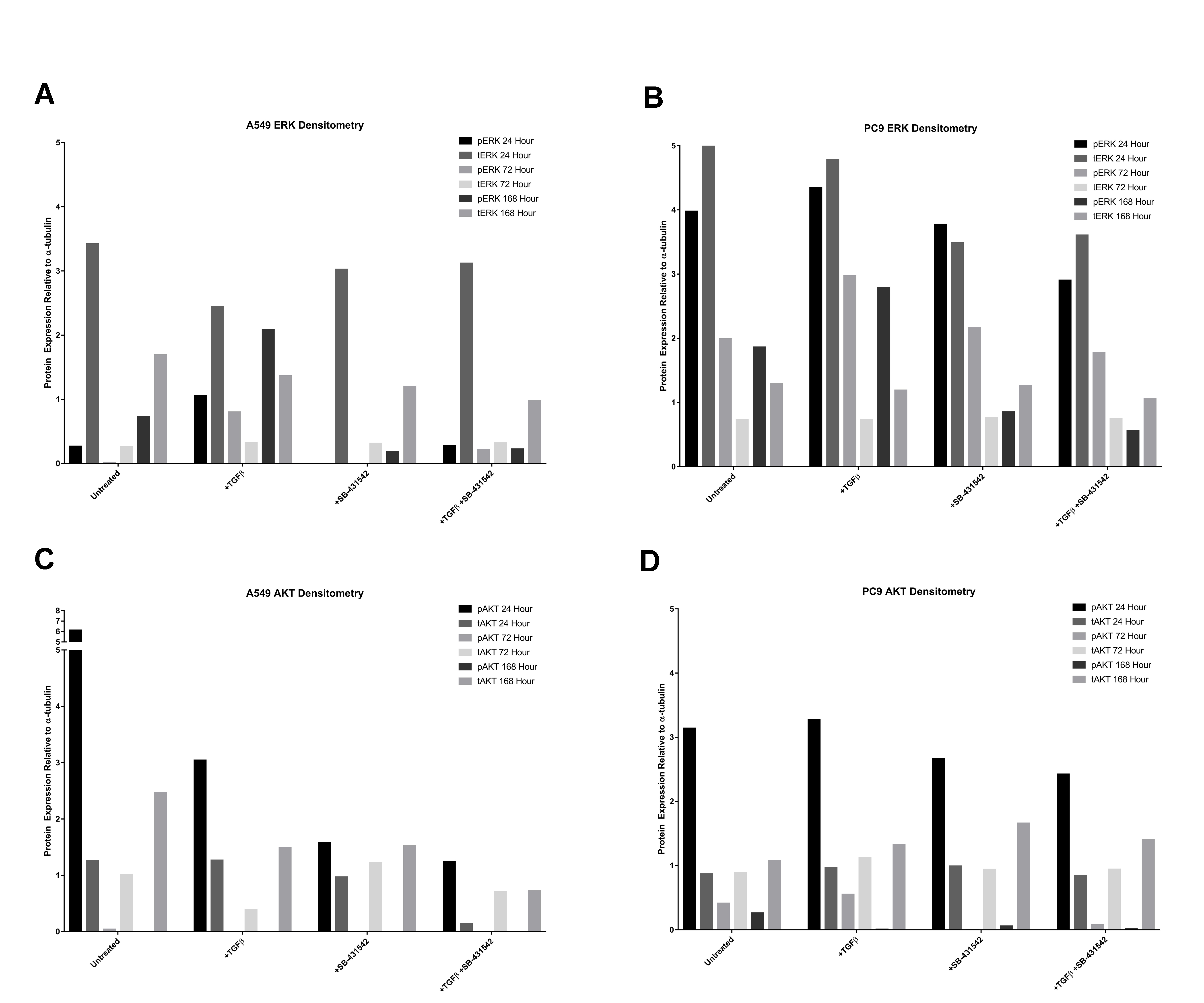
**

***Supplementary Figure 8: Densitometry of Figure 6 western blots.* (A)** pERK and tERK in A549 **(B)** pERK and tERK in PC9 **(C)** pAKT and AKT in A549 **(D)** pAKT and AKT in PC9. Blots were quantified using ImageJ and quantification was calculated using the area under the curve measurements for each band using the same sized box sample for each to ensure consistency (n=1).

**Supplementary Figure 9**


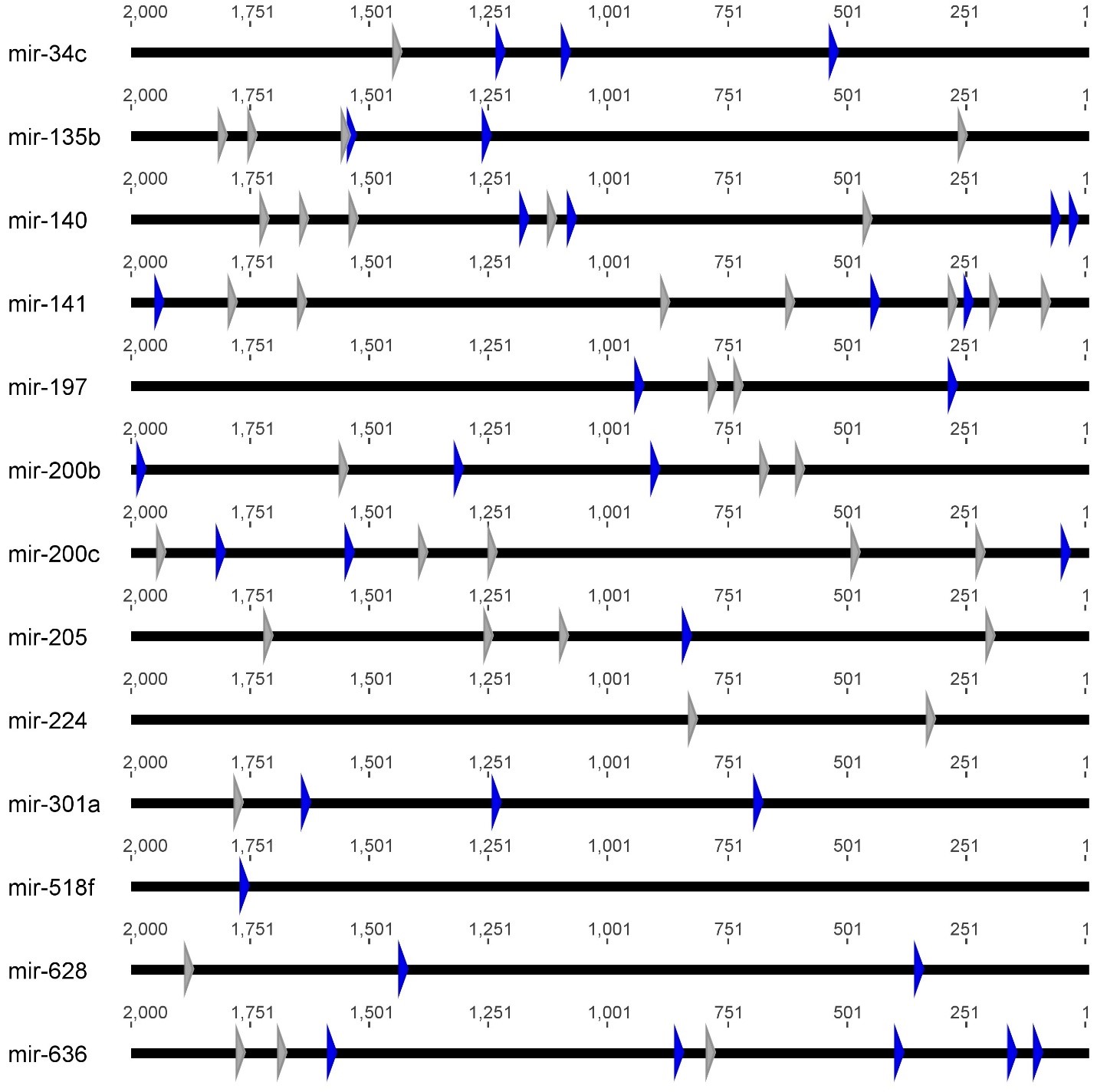


***Supplementary Figure 9: Signature microRNA genes contain both putative SBE elements and putative ELK1 binding sites.*** Promoter analysis was conducted using the ChipMAPPER algorithm [1]. Twelve out of 13 of the signature microRNA genes contain putative SBE elements as represented by the grey triangle with conservative E-values less than or equal to 25 and a score greater than 3.0. Twelve out of the 13 signature miRNA also contain putative ELK1 binding sites meting the same inclusion criteria as represented by the blue triangles.

1. Marinescu, V.D., I.S. Kohane, and A. Riva, *The MAPPER database: a multi-genome catalog of putative transcription factor binding sites.* Nucleic Acids Research, 2005. **33**(suppl 1): p. D91-D97.
2. Koizumi, F., et al., *Establishment of a human non-small cell lung cancer cell line resistant to gefitinib.* Int J Cancer, 2005. **116**(1): p. 36-44.
